# Comparative performance evaluation of QIAreach QuantiFERON-TB and tuberculin skin test for diagnosis of tuberculosis infection in Viet Nam

**DOI:** 10.1038/s41598-023-42515-1

**Published:** 2023-09-14

**Authors:** Luan Nguyen Quang Vo, Thi Thu Phuong Tran, Hai Quang Pham, Han Thi Nguyen, Ha Thu Doan, Huyen Thanh Truong, Hoa Binh Nguyen, Hung Van Nguyen, Hai Thanh Pham, Thuy Thi Thu Dong, Andrew Codlin, Rachel Forse, Tuan Huy Mac, Nhung Viet Nguyen

**Affiliations:** 1 Friends for International TB Relief, 6th Floor, 1/21 Le Van Luong St., Nhan Chinh Ward, Thanh Xuan District, Ha Noi, Viet Nam; 2 Department of Global Public Health, Karolinska Institutet, WHO Collaboration Centre on Tuberculosis and Social Medicine, Stockholm, Sweden; 3 National Lung Hospital, 463 Hoang Hoa Tham, Vinh Phuc, Ba Dinh, Ha Noi, Viet Nam; 4 Hai Phong Lung Hospital, Tran Tat Van, Trang Minh, Kien An, Hai Phong, Viet Nam; 5 University of Medicine and Pharmacy, Vietnam National University, Ha Noi, Viet Nam

**Keywords:** Tuberculosis, Diagnosis

## Abstract

Current WHO-recommended diagnostic tools for tuberculosis infection (TBI) have well-known limitations and viable alternatives are urgently needed. We compared the diagnostic performance and accuracy of the novel QIAreach QuantiFERON-TB assay (QIAreach; index) to the QuantiFERON-TB Gold Plus assay (QFT-Plus; reference). The sample included 261 adults (≥ 18 years) recruited at community-based TB case finding events. Of these, 226 underwent Tuberculin Skin Tests and 200 returned for interpretation (TST; comparator). QIAreach processing and TST reading were completed at lower-level healthcare facilities. We conducted matched-pair comparisons for QIAreach and TST with QFT-Plus, calculated sensitivity, specificity and area under a receiver-operating characteristic curve (AUC), and analyzed concordant-/discordant-pair interferon-gamma (IFN-γ) levels. QIAreach sensitivity and specificity were 98.5% and 72.3%, respectively, for an AUC of 0.85. TST sensitivity (53.2%) at a 5 mm induration threshold was significantly below QIAreach, while specificity (82.4%) was statistically equivalent. The corrected mean IFN-γ level of 0.08 IU/ml and corresponding empirical threshold (0.05) of false-positive QIAreach results were significantly lower than the manufacturer-recommended QFT-Plus threshold (≥ 0.35 IU/ml). Despite QIAreach’s higher sensitivity at equivalent specificity to TST, the high number of false positive results and low specificity limit its utility and highlight the continued need to expand the diagnostic toolkit for TBI.

## Introduction

Tuberculosis (TB) is one of the world’s leading causes of preventable death, with approximately 1.6 million TB-related mortalities in 2021^1^. An estimated 1.7–2.3 billion persons are infected with *M. tuberculosis,* of whom about 5–15% will progress to active TB^2^. This reservoir could sustain TB incidence and mortalities for decades to come, even if all transmission were to cease from today onwards^3^. Therefore, prompt diagnosis and treatment of TB infection (TBI) constitutes a core component of the WHO’s End TB strategy^4^.

Viet Nam ranked 11 out of the 30 high TB burden countries (HBC) in 2021^5^. The measured annual risk of TBI is 1.7% and an estimated 30% of the population likely has a TBI, which increases to over 40% in densely populated urban areas^6^. As Viet Nam has codified its ambitions to end TB^7^, the country has committed in the UN High-Level Meeting targets to actively address this pool of infections by treating 343,390 individuals with TBI^8^. However, similar to other HBCs, Viet Nam is not on track to meet this target, in part due to major gaps in its diagnostic capacity and supply^9^.

Currently, TBI cannot be directly detected due to its latency, but instead relies on host response-based immunodiagnostics. In Viet Nam, Tuberculin Skin Tests (TST) using Purified Protein Derivative (PPD) Tuberculin Mammalian Bulbio (PPD-Bulbio; BB-NCIPD Ltd., Sofia, Bulgaria) is the preferred diagnostic tool for TBI of the National TB Control Program (NTP) over Interferon-Gamma Release Assays (IGRA)^10^. However, both require a competent immune response to identify individuals infected with TB and remain imperfect diagnostic options due to the inability to gauge the likelihood of progression to active disease^11,12^. The challenges and shortcomings of both have been well-documented, so that it is vital that new diagnostic tools and methods are developed that can sustain their advantages while addressing their limitations^13,14^. Numerous studies have demonstrated the superior diagnostic performance of the latest-generation IGRA, QuantiFERON-TB Gold Plus (QFT-Plus; QIAGEN GmbH, Hilden, Germany), which assesses both a CD4 and a CD8 T-cell response to diagnose TBI with greater diagnostic accuracy^15–17^. Despite the improved performance, uptake is hindered by complex specimen handling, sophisticated laboratory requirements and high direct costs^14,18^. Moreover, studies have shown similarly poor performance of both TST and IGRA in their predictive value of prognosing progression to active TB^19,20^.

Recently a new lateral flow immunoassay was developed with the aim to provide prompt and reliable diagnosis, even in challenging and remote settings. The QIAreach QuantiFERON-TB assay (QIAreach; QIAGEN GmbH, Hilden, Germany) is a semi-automated lateral flow immunoassay that emulates the QFT-Plus mechanism by measuring the level of interferon-gamma (IFN-γ) in plasma released in CD4 and CD8 T-cells utilizing a single blood collection tube (BCT) which matches the QTF-Plus TB2 tube. However, instead of requiring the complex Enzyme-linked immunoassay (ELISA) method for IFN-γ detection, QIAreach relies on digital fluorescence lateral flow nanoparticle technology that enables the compression of the IFN-γ detection step into a single cartridge (eStick) processed on a portable platform (eHub) that provides a binary result (positive/negative) within 20 min^21^. Our study evaluated the diagnostic performance and accuracy of QIAreach versus QFT-Plus and compared it with TST.

## Results

### Sample characteristics

The study screened 360 individuals attending the community-based active TB case finding events. Eighty-two individuals did not meet the study’s eligibility criteria. Among the 278 participants recruited for the study, there were 17 indeterminate results on QFT-Plus, which were excluded from the performance analysis. We encountered no adverse events from administration of the index, reference or comparator tests. The final sample included 261 participants, of whom 62.8% were female (164/261) (Table 1) with a median age of 61 years (Interquartile range: 54–67). About 49.8% (130/261) of participants presented with a cough, 33.3% (87/261) with fatigue, 28.0% (73/261) with chest pain, and 24.5% (64/261) with dyspnea. The proportion reporting a past COVID-19 infection was 49.4% (129/261), while 6.1% (16/261) reported a history of TB. The proportion of participants reporting comorbid diabetes was 12.6% (33/261) and 8.1% (21/261) indicated concomitant use of immunosuppressants. About 23.4% (61/261) of participants used tobacco habitually.

**Table 1:** Sample characteristics.

	Total, n (% total)	QFT-Plus positive, n (% positive)	QIAreach positive, n (% positive)	p-value^¥^
Total	261 (100)	66 (25.3)	119 (45.6)	
Gender				
Male	97 (37.2)	29 (29.9)	49 (50.5)	< 0.001
Female	164 (62.8)	37 (22.6)	70 (42.7)	< 0.001
Age group				
Under 45	34 (13.0)	5 (14.7)	11 (32.4)	0.014
45–59	73 (28.0)	22 (30.1)	34 (46.6)	< 0.001
60–79	134 (51.3)	36 (26.9)	67 (50.0)	< 0.001
80 and above	20 (7.7)	3 (15.0)	7 (35.0)	0.046
Median age (IQR)	61 (54–67)	60.5 (56–67)	62 (56–67)	
Social Health Insurance	238 (91.2)	57 (23.9)	104 (43.7)	< 0.001
Habitual tobacco use	61 (23.4)	14 (23.0)	32 (52.5)	< 0.001
Diabetes	33 (12.6)	14 (42.4)	17 (51.5)	0.083
History of TB	16 (6.1)	8 (50.0)	10 (62.5)	0.157
Use of immunosuppressants	21 (8.1)	4 (19.0)	8 (38.1)	0.046
Contact with TB patient	15 (5.8)	4 (26.7)	8 (53.3)	0.046
History of COVID-19 infection				
No COVID-19 infection	132 (50.6)	34 (25.8)	64 (48.5)	< 0.001
1–2 months ago	31 (11.9)	9 (29.0)	14 (45.2)	0.025
3 months ago	86 (32.9)	20 (23.3)	36 (41.9)	< 0.001
4–5 months ago	12 (4.6)	3 (25.0)	5 (41.7)	0.157
Tuberculosis symptoms				
Cough	131 (50.2)	29 (22.1)	55 (42.0)	< 0.001
Fatigue	87 (33.3)	21 (24.1)	36 (41.4)	< 0.001
Chest pain	73 (28.0)	20 (27.4)	36 (49.3)	< 0.001
Shortness of breath	64 (24.5)	13 (20.3)	32 (50.0)	< 0.001
Loss of appetite	27 (10.3)	10 (37.0)	15 (55.6)	0.025
Unintentional weight loss	22 (8.4)	8 (36.4)	13 (59.1)	0.025
Night sweats	17 (6.5)	10 (58.8)	15 (88.2)	0.025
Fever	5 (1.9)	2 (40.0)	3 (60.0)	0.564

Descriptive statistics of demographic and clinical participant covariates with age reported as median with the interquartile range. Mann–Whitney U-test and Chi-squared tests were used to calculate p-values; p < 0.05 was considered statistically significant.

^¥^McNemar’s test used to compare QFT-Plus with QIAreach within each subgroup.

### Diagnostic performance of QIAreach

QFT-Plus positivity in the sample was 25.3% (66/261) compared to a QIAreach positivity of 45.6% (119/261). The rate of concordance between QIAreach and QFT-plus was 78.9% (206/261) (Cohen’s κ = 0.559; p < 0.001), including 65 concordant positive and 141 concordant negative results (Table 2). Only 1.5% (1/66) of participants with a positive QFT-Plus result had a false-negative QIAreach result, but 27.7% (54/195) of participants tested positive with QIAreach and negative with QFT-Plus (i.e., false positive). The disagreement between the QIAreach and QFT-Plus results was statistically significant (p < 0.001).

**Table 2:** QIAreach, QFT-Plus and TST results.

	Total, n (%)	TST (5 mm) positive, n (%)	TST (5 mm) negative, n (%)	p-value^¥^	TST (10 mm) positive, n (%)	TST (10 mm) negative, n (%)	p-value^¥^
QIAreach	200 (100.0)						
Positive	84 (42.0)	33 (39.3)	51 (60.7)	< 0.001	16 (19.1)	68 (81.0)	< 0.001
Negative	116 (58.0)	19 (16.4)	97 (83.6)		7 (6.0)	109 (94.0)	
QFT-Plus	200 (100.0)						
Positive	47 (23.5)	25 (53.2)	22 (46.8)	0.475	15 (31.9)	32 (68.1)	< 0.001
Negative	153 (76.5)	27 (17.7)	126 (82.4)		8 (5.2)	145 (94.8)	

Contingency tables of QIAreach and TST results compared to the reference standard. TST results were presented at 5 mm and 10 mm induration thresholds. Chi-squared tests were used to calculate p-values; p < 0.05 was considered statistically significant.

^¥^McNemar’s test used to compare QIAreach and QFT-Plus with TST at a threshold of 5 mm and 10 mm.

Across the full sample, QIAreach had a sensitivity of 98.5% (95% CI [91.8%; 100.0%]) and specificity of 72.3% (95% CI [65.5%; 78.5%]) (Table 3). With respect to diagnostic accuracy, we measured an AUC index of 0.85 (95% CI [0.82; 0.89]).

**Table 3:** Sensitivity, specificity and ROC AUC of QIAreach.

	Score [95% CI]
Sensitivity	98.5 [91.8; 100.0]
Specificity	72.3 [65.5; 78.5]
Positive predictive value	54.6 [45.2; 63.8]
Negative predictive value	99.3 [96.1; 100.0]
ROC AUC	0.85 [0.82; 0.89]

Sensitivity, specificity, positive predictive value, negative predictive value, Area under the Receiver Operating Charactistics curve (ROC AUC) of QIAreach were calculated using manufacturer instructions with QFT-Plus as the reference standard (n = 261).

### Comparative evaluation of QIAreach and TST

**Figure 1:**
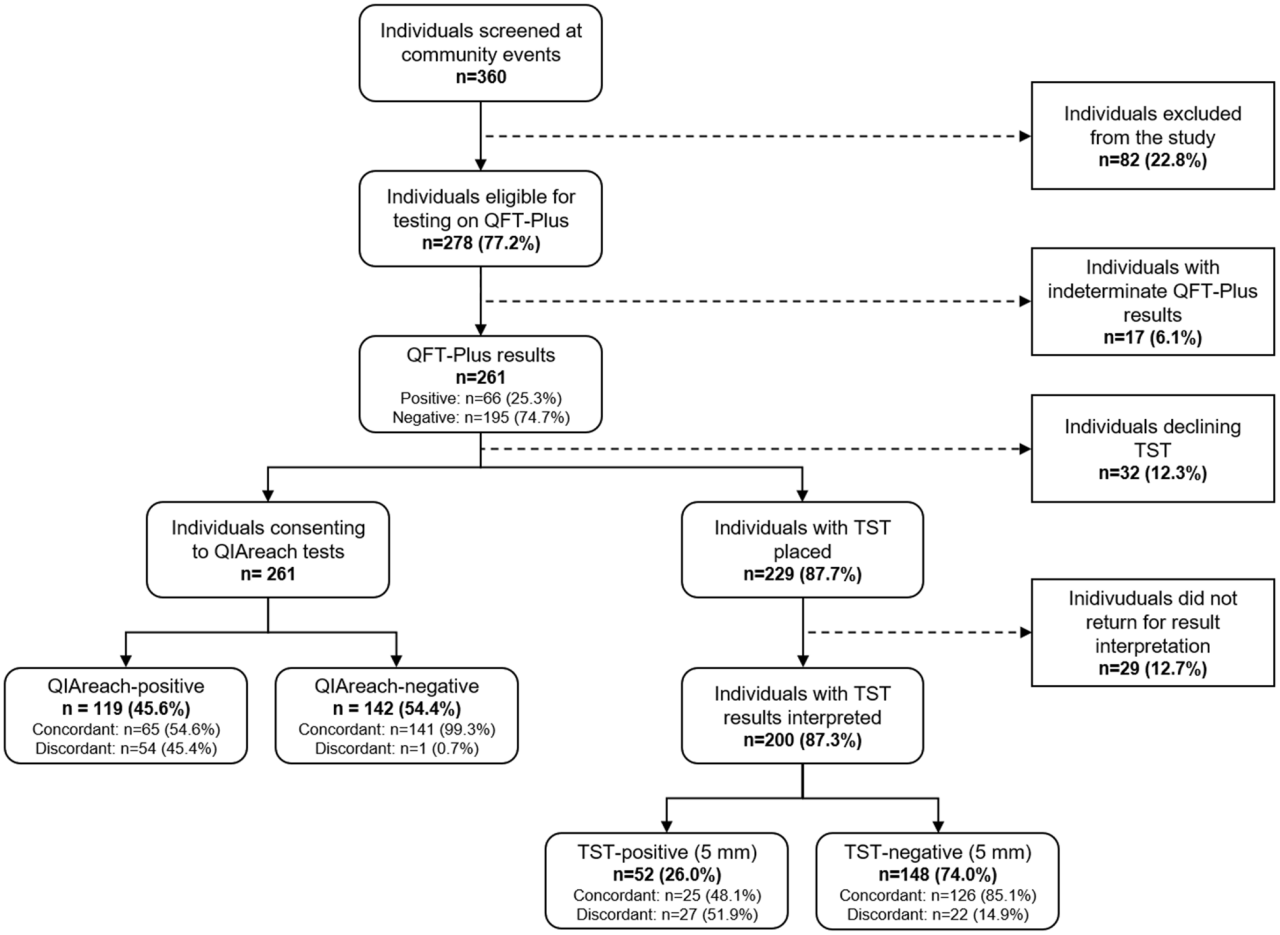
Study flow diagram. Cascade of participants including reasons for drop-off such as not meeting inclusion criteria, receiving indeterminate QFT-Plus results, declining to receive a TST and failing to present for result interpretation.

Of the 261 participants, 23.4% (61/261) declined to participate in a TST or did not return for TST interpretation. Of the remaining 200 individuals with a TST result, the rate of participants with a positive QFT-Plus result was 23.5% (47/200) (Fig. 1). Approximately 26.0% (52/200) were evaluated as TST-positive using an induration threshold of 5 mm (Table 2). The overall concordance rate between TST and QFT-plus was 75.5% (151/200). The false negative rate was 46.8% (22/47), while the rate of false positives was 17.7% (27/153). At a 10 mm TST threshold, the number of true negative and false negative results increased, improving the overall concordance rate to 80.0% (160/200). The rate of false positives declined to 5.2% (8/153), while the false negative rate increased to 68.1% (32/47).

Within the subsegment of 200 participants with matched results for QIAreach, TST and QFT-Plus, the QIAreach sensitivity was 97.9%, which was significantly higher than the TST sensitivity using both 5 mm (53.2%) and 10 mm (31.9%) induration thresholds (both p < 0.001) (Table 4). Meanwhile, the QIAreach specificity was statistically equivalent compared to the specificity of TST using the 5 mm threshold (75.2% vs. 82.4%; p = 0.139), but significantly lower when using the 10 mm threshold (75.2% vs. 94.8%; p < 0.001). Similarly, the QIAreach AUC was significantly higher than the TST AUC at 5 mm (0.87 vs. 0.68; p < 0.001) and 10 mm induration thresholds of (0.87 vs. 0.63; p < 0.001).

**Table 4:** Sensitivity, specificity and concordance of QIAreach compared to TST in individuals with a valid TST result (n = 200).

	QIAreach Score [95% CI]	TST (5 mm) Score [95% CI]	p-value	TST (10 mm) Score [95% CI]	p-value
Sensitivity	97.9 [88.7; 99.9]	53.2 [38.1; 67.9]	< 0.001	31.9 [19.1; 47.1]	< 0.001
Specificity	75.2 [67.5; 81.8]	82.4 [75.4; 88.0]	0.139	94.8 [90.0; 97.7]	< 0.001
Positive predictive value	54.8 [43.5; 65.7]	48.1 [34.0; 62.4]	0.467	65.2 [42.7; 83.6]	0.382
Negative predictive value	99.1 [95.3; 100.0]	85.1 [78.4; 90.4]	< 0.001	81.9 [75.4; 87.3]	< 0.001
ROC AUC	0.87 [0.83; 0.91]	0.68 [0.60; 0.76]	< 0.001	0.63 [0.56; 0.70]	< 0.001

Sensitivity, specificity, positive predictive value, negative predictive value, AUC of Tuberculin skin test (TST) with 5 mm and 10 mm induration threshold values for the subset of individuals with a TST result with QFT-Plus as reference standard (n = 200). Chi-squared tests were used to calculate p-values; p < 0.05 was considered statistically significant.

### Analysis of IFN-γ levels by QIAreach and QFT-Plus results

The corrected (TB2-Nil) and uncorrected (TB2) mean IFN-γ levels in participants identified by QIAreach as false positives were 0.079 IU/ml (95% CI [0.043; 0.114]) and 0.317 IU/ml (95% CI [0.207; 0.428]), respectively (Table 5). Similarly, the empirical optimal threshold values for TB2-Nil and TB2 for evaluation of QIAreach-positivity were 0.05 and 0.18, respectively, both of which were significantly lower than the manufacturer’s recommended threshold for QFT-Plus (≥ 0.35 IU/ml). Conversely, among those who returned a positive result with QFT-Plus, only one participant was negative with QIAreach, i.e., a false negative, with TB2 and TB2-Nil values of 0.57 and 0.47 IU/mL, respectively.

**Table 5:** Analysis of IFN-γ levels by QIAreach and QFT-Plus results.

		IFN-γ level [95% CI]	Empirical estimation of threshold	Theoretical sensitivity at empirical threshold	Theoretical specificity at empirical threshold
Corrected (TB2-Nil)	Total	0.475 [0.322; 0.629]			
	False positive	0.079 [0.043; 0.114]			
	False negative*	0.470 [–; –]			
	QIAreach positive^†^	1.021 [0.709; 1.332]	0.05	0.81	0.85
	QIAreach negative^¶^	0.019 [0.007; 0.030]			
	QFT-Plus positive^†^	1.783 [1.290; 2.276]	0.27	0.94	0.98
	QFT-Plus negative^¶^	0.033 [0.020; 0.045]			
Uncorrected (TB2)	Total	0.750 [0.550; 0.951]			
	False positive	0.317 [0.207; 0.428]			
	False negative*	0.570 [–; –]			
	QIAreach positive^ǂ^	1.530 [1.130; 1.931]	0.18	0.82	0.92
	QIAreach negative^¥^	0.095 [0.078; 0.112]			
	QFT-Plus positive^ǂ^	2.508 [1.880; 3.136]	0.41	0.95	0.95
	QFT-Plus negative^¥^	0.154 [0.119; 0.189]			
Nil	Total	0.275 [0.203; 0.347]			
	False positive	0.239 [0.122; 0.356]			
	False negative*	0.100 [–; –]			
	QIAreach positive^¤^	0.512 [0.365; 0.659]	0.10	0.72	0.80
	QIAreach negative^±^	0.076 [0.063; 0.090]			
	QFT-Plus positive^¤^	0.730 [0.492; 0.967]	0.15	0.77	0.82
	QFT-Plus negative^±^	0.121 [0.087; 0.156]			

Corrected (TB2-Nil) and uncorrected (TB2) mean IFN-γ concentrations in IU/ml. Includes 95% confidence intervals, empirical estimation of threshold, and theoretical sensitivity and specificity at the empirical threshold. Student’s T-tests were applied to compare the means of two groups; p < 0.05 was considered statistically significant.

*Only one participant had a false negative result.

^†^Comparison between TB2-Nil value of QIAreach-positive and QFT-Plus-positive: p = 0.008.

^¶^Comparison between TB2-Nil value of QIAreach-negative and QFT-Plus-negative: p = 0.114.

^**ǂ**^Comparison between TB2 value of QIAreach-positive and QFT-Plus-positive: p = 0.008.

^**¥**^Comparison between TB2 value of QIAreach-negative and QFT-Plus-negative: p = 0.008.

^¤^Comparison between Nil value of QIAreach-positive and QFT-Plus-positive: p = 0.110.

^±^Comparison between Nil value of QIAreach-negative and QFT-Plus-negative: p = 0.037.

The corrected mean IFN-γ level in QFT-Plus-positive participants (1.783 IU/ml; 95% CI [1.290; 2.276]; p = 0.008) was significantly higher than in QIAreach-positive participants (1.021 IU/ml; 95% CI [0.708; 1.332]). The corrected mean IFN-γ levels in QFT-Plus- and QIAreach-negative participants were not significantly different (0.033 IU/ml; 95% CI [0.020; 0.045] vs. 0.019 IU/ml; 95% CI [0.007; 0.030]; p = 0.114).

## Discussion

Our study found that QIAreach had a high sensitivity when compared to QFT-Plus (reference standard). In direct comparison with TST using a 5 mm induration threshold, the assay also performed well, achieving a significantly higher sensitivity with a statistically equivalent specificity. However, the assay produced a high number of false positive results, which resulted in a significantly lower specificity compared to TST using a 10 mm induration threshold, which was the standard of care for the majority of persons with TBI according to national guidelines in Viet Nam at the time of the study^22^.

The assay’s high sensitivity was concordant with recent studies that reported similar high sensitivity versus QFT-plus ranging from 99.1 to 100%^23–25^. Moreover, QIAreach displayed a high diagnostic performance in our study, which may qualify the assay as sufficiently accurate for a diagnostic test^26^. As studies have shown a better predictive ability of IGRAs than TSTs, the level of concordance with QFT-Plus and diagnostic accuracy suggests QIAreach’s potential as an additional tool for TBI diagnosis at least based on its ability to accurately identify persons with TBI^27^.

To our knowledge, this was the first study to include a matched comparison between QIAreach and TST. The latter currently represents the programmatic standard for TBI diagnosis in Viet Nam as well as many other HBCs^28^. However, there is urgent need to validate new TBI diagnostic tools following the detection of substantial variability in TST positivity despite sourcing the tuberculin from the same manufacturer (PPD-Bulbio)^10^. This variability has been reflected in a decline in positivity and subsequent increase in resource requirements for meeting national TPT targets^29^. In response, the Viet Nam NTP issued new rapid guidance in September 2022 to lower the positivity threshold to 5 mm, and improve positivity and programmatic scale-up of TPT^30^. Thus, our analysis plan included both 10 mm and 5 mm induration sizes to accommodate the latest developments with the former being the standard of care at the time of the study. As the NTP has shifted its focus towards the more aggressive strategy of higher sensitivity in exchange for lower specificity, in the scenario of a 5 mm threshold our results show that QIAreach may serve as a viable clinical alternative to TST.

This is also one of the first published studies to validate QIAreach outside of the health facility setting as per its intended purpose of expanding access to IGRA closer to the point of care. Operationally, QIAreach offers many of the similar key advantages of QFT-Plus compared to TST. Only one visit is required to complete the test, while TST still requires one visit for testing and a second for interpretation^31,32^. In our research, almost a quarter of recruited participants did not agree to conduct the test or did not present for results interpretation. These data exemplify the hindrance created by the inconvenience of the TST. Clinically, the T-Cell-based QIAreach offers protection from the array of confounders commonly affecting TST results such as age, nutrition, immunology, genetics, BCG vaccination, and cross-reactivity with non-tuberculous mycobacteria^33^.

Simultaneously, QIAreach also aims to resolve key challenges of the QFT-Plus assay by offering simplified operating procedures, faster turnaround time, and greater flexibility of deployment. QFT-Plus requires a laboratory infrastructure, technical expertise, and expensive equipment, while the QIAreach eStick-eHub architecture aims to emulate the success of other cartridge-based diagnostic tools for TB in low-resource settings that have little to no access to sophisticated laboratory capacity. The rapid turnaround time that characterizes the eStick-eHub design also enables higher throughput of up to 24 samples per eHub per hour^34^.

Nevertheless, our study also exposed key performance issues in our setting that will need to be evaluated further to facilitate uptake of this new tool. QFT-Plus needs four BCTs (TB1, TB2, Nil and mitogen) to optimize results interpretation. Meanwhile, QIAreach relies only on a single BCT, which is designed to maximize sensitivity with antigens optimized to stimulate CD4 and CD8 T-cells. Past studies have found this method to possibly increase IFN-γ levels which would inflate the number of results considered positive^17,35^. These factors may have contributed to the low specificity of QIAreach observed on this study as evinced in the analysis of uncorrected (TB2) and corrected (TB2-Nil) mean IFN-γ levels. Specifically, the uncorrected and corrected IFN-γ levels were 1.6–1.7 times higher in participants testing negative on QFT-Plus than on QIAreach. Thus, the absence of the negative (Nil) control likely contributed to the impaired specificity, especially if QIAreach-positivity was calibrated using QFT-Plus thresholds.

The lower specificity on our study was discordant with the limited available evidence base. Two hospital-based studies in Italy and Japan detected high concordance between QIAreach and QFT-Plus performance and specificity in particular. The Italian study observed a specificity versus QFT-Plus of 93.4% and an overall concordance with QFT-Plus of 95.7% (κ = 0.96) among 130 persons with confirmed TB and 174 healthy controls^23^. Similarly, the Japanese study conducted in 41 persons with active TB and 42 healthy individuals recorded a specificity of 97.6% among the TB patient cohort with an overall concordance of 98.8% versus QFT-Plus in the sample (κ = 0.98)^24^. Moreover, the study highlighted that the IFN-γ concentration cutoff point for QIAreach was similar to that of the QFT-Plus assay (0.35 IU/mL) for the active TB population^24^, which our study did not corroborate. Based on these data, a hypothesis to explain the low specificity of QIAreach compared QFT-Plus may be the study setting. Contrary to these two examples, our study recruited participants in the community with a comparatively lower rate of TB infection and disease. This setting may also be exposed to confounding and bias reflected in the greater variance in the QFT-Plus results as seen in the high indeterminate rate (17/278 = 6.1%).

It is evident that more work is needed to specify the utility and role that QIAreach can play in the global scale-up of TPT. Studies should also incorporate economic and market analyses once the product moves towards commercialization to address the most common criticism of IGRAs—their costs^36^—as health economic analyses have estimated IGRAs to be more cost-effective than TST^37^. For now, the test is not recommended in national guidelines. At the current diagnostic accuracy, it may only find limited application in priority groups with elevated risk of progression and settings where the benefits of aggressive intervention outweigh the health system and patient costs of unnecessary treatment. An example of such a priority group may consist of household and close contacts of MDR-TB patients as a recent study reported a strong correlation between results from TST and QFT-Plus when detecting TBI in MDR-TB contacts, concluding TST could be used in place of QFT-Plus^38^.

Our study was limited in a number of ways that may affect its generalizability. The aforementioned lack of a formal health economic analysis prevents the ability to build an investment case for policymakers and multilateral funding agencies. Another key limitation of the study was a lack of comparison between QIAreach and bacteriologically-confirmed individuals with TB, including children, which precluded the determination of “true” sensitivity and specificity of the assay in Viet Nam. In addition, large gaps in the TBI cascade for TST resulted in a smaller sample size, which may have deleteriously affected the statistical comparison between QIAreach and TST resulting in their respective specificities showing no significant difference at a 5 mm threshold. These drops in the cascade may have also introduced bias into the results.

## Conclusions

Currently, TST and IGRA are the only recommended diagnostics for TBI. However, the limitations of these methods still impair the accuracy, effectiveness and uptake of these tools and scale-up of TPT overall. This study provided evidence on the performance and accuracy of QIAreach assay compared to TST and QFT-Plus. Our results showed high sensitivity and AUC classification, but also exposed a suboptimal specificity, thereby potentially affecting its value and utility, particularly in low-resource, high-burden settings. Thus, more evidence for this new IGRA assay and other new diagnostic tools for TBI remain urgently needed. Nevertheless, this study was among the first to evaluate QIAreach along several dimensions and contributes to the available evidence base to inform future research, programmatic implementation and policy development towards reducing the global seedbed of TB.

## Material and methods

### Study design and aims

This was a prospective, unblinded, cross-sectional study to evaluate the comparative diagnostic performance, i.e., sensitivity, specificity, and accuracy of QIAreach (index test) against TST (comparator) for TBI diagnosis in community and lower healthcare settings. QFT-Plus served as the reference standard, so that sensitivity and specificity reported in this study represent positive and negative percent agreement versus the reference.

### Study setting and participants

Participants were recruited consecutively at community-based active TB case finding (ACF) events in Da Phuc commune, Duong Kinh district, Hai Phong province, Viet Nam in April 2022. These events have been described in greater detail elsewhere^39,40^.

Eligible persons were aged 18 or older, provided informed written consent to participate and met at least one of the following inclusion criteria: (i) documented exposure to an index person with TB, (ii) comorbid HIV (irrespective of antiretroviral therapy); comorbid diabetes mellitus, (iii) being homeless or illicit drug users, (iv) reported limited access to health services due to socioeconomic conditions or otherwise, and v) had TB symptoms.

### Sample size

We calculate the sample size to power a matched-pair comparison of proportions using McNemar’s Z-test for 1-sided equality between QIAreach and QFT-Plus. Our confusion matrix assumed an overall concordance of 94% with false positive and false negative rates of 5% and 1%, respectively, based on published positive and negative percent agreement rates^24,25,41^. With a confidence level of α = 95% and a power of β = 80%, the estimated sample size was n = 230. We included a 20% contingency for attrition, data losses and post-hoc exclusion for a final sample size of n = 276.

### Specimen and data collection

Whole blood samples were collected and monitored following standard biosafety procedural controls. At noon and at the end of each day, blood samples were transported to the laboratories. Each participant would be tested with both QIAreach and QFT-Plus following manufacturer instructions. Samples were collected in five blood collection tubes (BCT), including four for QFT-Plus (Nil, TB1, TB2, Mitogen) and one for QIAreach (TB2). All blood specimens were collected prior to administration of TST as recommended. All laboratory staff were trained by experts from the National Reference Laboratory which is located at the National Lung Hospital in Ha Noi. Demographic information and clinical history were also obtained from patients through interviews.

### Testing procedures

Peripheral blood sampling and processing for QIAreach and QFT-Plus were performed and interpreted according to manufacturer instructions. The tubes were incubated at 37 °C ± 1 °C for 16–24 h and then placed in a centrifuge at 2000–3000*g* for 15 min. Plasma harvested from a QIAreach tube was deposited into an eStick on-site, while plasma from the four QFT-plus tubes was packaged and transported to the provincial lung hospital laboratory for processing via the Enzyme-linked immunosorbent assay method. Qualitative QIAreach results were evaluated after 20 min of processing on the eHub. QFT-Plus results were returned 2–3 days after sample collection. QFT-Plus results were presented as IU/mL of INF-γ levels and interpreted in accordance to manufacturer instructions^42,43^.

TSTs were administered using standardized PPD-Bulbio at 5 TU/0.1 mL by trained government health staff at the community TB screening events. Participants were invited to present for interpretation at the district health center between 48 and 72 h after administration of the test and results were recorded as exact induration size (mm). The TST-positivity threshold was 10 mm in accordance to contemporary national guidelines.

### Statistical analyses

We calculated descriptive statistics for the sample and created contingency tables to assess the diagnostic performance measurements, including sensitivity, specificity, and associated 95% confidence intervals for QIAreach and TST in comparison with the reference standard. We calculated AUCs, i.e., areas under the Receiver Operating Characteristics (ROC) curve, to estimate the comparative diagnostic accuracy of the index, comparator and reference assays. Given the high rate of false positives encountered, a post-hoc analysis assessed associations between QIAreach results and the corrected (TB2-Nil) and uncorrected (TB2) IFN-γ levels. Additional post-hoc analyses included description of time-to-result by QIAreach result and fitting multivariate logistic regression models to identify patient covariates associated with discordance between QIAreach and QFT-Plus. The results of these latter two analyses have been included in the supplementary information S1. Statistical comparison utilized appropriate tests such as McNemar’s Test for matched-pair comparisons, chi-squared for multiple proportions, Student’s T-test for means and non-parametric tests such as the Mann–Whitney for medians of data deviating from the Gaussian distribution. Missing data were retrieved from participants post-hoc or, if unsuccessful, excluded from the analysis. A p-value of < 0.05 was considered statistically significant. All analyses were performed on Stata v14 (StataCorp, College Station, TX, USA).

### Ethical considerations

The study was conducted in accordance with the Helsinki Declaration (7th Revision). Ethical approvals were obtained from the National Lung Hospital Institutional Review Board (46/20/CN-HĐĐĐ) and Ministry of Health Scientific and Ethics Committee (218/CN-HĐĐĐ). All participants provided informed, written consent and were able to opt in to evaluate QIAreach, TST or both. Non-participation did not affect the provision of care. All data were anonymized prior to analysis.

### Supplementary Information

Supplementary Information.

## Data Availability

The data that support the findings of this study are available from the Viet Nam National TB Control Program and the Hai Phong Provincial Lung Hospital, but restrictions apply to their availability. The study protocol and data can be furnished upon reasonable request and with permission from the authors and of the Viet Nam National TB Control Program and Hai Phong Provincial Lung Hospital, respectively.
