# Supplementary material for: Comparative performance evaluation of QIAreach QuantiFERON-TB and tuberculin skin test for diagnosis of tuberculosis infection in Viet Nam
Source: Sci Rep. 2023 Sep 14;13:15209. doi: 10.1038/s41598-023-42515-1 (PMC10502094; doi:10.1038/s41598-023-42515-1)
Supplement: Supplementary file 1 — Supplementary Information. [file 41598_2023_42515_MOESM1_ESM.docx]

# Supplementary methods

In terms of QFT-Plus interpretation (Supplementary Table S1), a positive result is defined as an antigen response in TB1 and/or TB2 of ≥ 0.35 IU/mL above the negative control and ≥25% of the negative control (LTBI). A negative result was recorded if the antigen response minus the negative control was <0.35 IU/mL or <25% of the negative control with a positive control (mitogen tube) of ≥0.5 IU/mL (no LTBI). A result was deemed indeterminate if the antigen tube minus the negative control was <0.35 IU/mL or <25% of the negative control with a positive control of <0.5 IU/mL or the antigen response of the negative control was >8 IU/mL.

**Supplementary Table S1: QFT-Plus result interpretation guidelines**


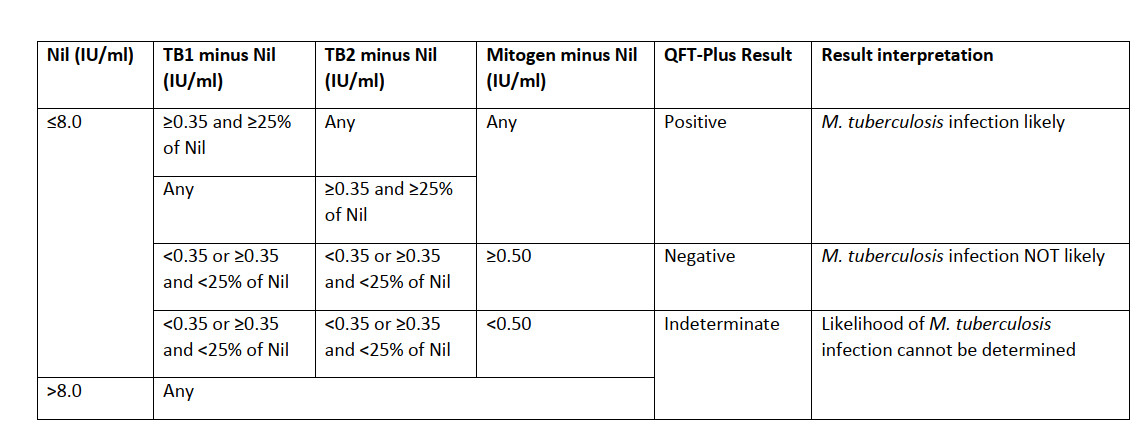


# Supplementary results

## QIAreach time-to-result analysis

Time-to-result was related to the level of fluorescent signals generated during the test, which is simultaneously a factor that could affect the accuracy of the QIAreach test. Prior studies have found that the higher the IFN- γ level of the sample, the shorter the time to result, and used IFN- γ concentration in plasma as a marker for the QIAreach assay in active TB. For this study, we examined the time to results for QIAreach with negative and positive QIAreach results and bifurcated by QFT-Plus result to understand the correlation of time and IFN-γ concentration (Supplementary Table S2). The median for all groups was 1,200 seconds (20 min). There was no statistical difference between samples with negative and positive QIAreach results and when bifurcated by QFT-Plus result. As such, the time to result did not prove to have a statistically significant correlation to IFN- γ level TB2.

**Supplementary Table S2. QIAreach time-to-result analysis**

|  | **Mean time (sec)** | **SD** | **Median** | **IQR** | **p-value** |
| --- | --- | --- | --- | --- | --- |
| **Total** | 1026.0 | 309.5 | 1200 | 960 - 1200 |  |
| **QIAreach** |  |  |  |  |  |
| Positive | 1024.5 | 309.9 | 1200 | 960 - 1200 | 0.966 |
| Negative | 1027.2 | 310.4 | 1200 | 930 - 1200 |  |
| **QFT-Plus** |  |  |  |  |  |
| Positive | 1062.5 | 278.5 | 1200 | 1200 - 1200 | 0.256 |
| Negative | 1013.6 | 319.1 | 1200 | 865 - 1200 |  |

## Association of risk factors with QIAreach results

QIAreach has a higher sensitivity and saves procedure time, which led us to consider whether risk factors could affect the sensitivity and specificity of QIAreach results. That is why we collected all participants' clinical history related to diabetes, TB history, TB symptoms, use of immunosuppressive drugs, cough, fever, and COVID-19. While we found a statistically significant association between shortness of breath and discordant QIAreach-QFT Plus results (p=0.017), the small sample size and limited clinical justification prevent meaningful conclusions (Supplementary Table S3).

**Supplementary Table S3.** **Regression analysis of patient covariates associated with discordance between QIAreach QFT TB and QFT-Plus results**

|  | **OR** | **p** | **95% CI** | **aOR** | **p** | **95% CI** |
| --- | --- | --- | --- | --- | --- | --- |
| **Gender** (Male vs Female) | 1.16 | 0.624 | 0.63; 2.14 | 0.70 | 0.481 | 0.26; 1.89 |
| **Age group** |  |  |  |  |  |  |
| Under 45 | Ref. |  |  | Ref. |  |  |
| 45 - 59 | 0.92 | 0.876 | 0.31; 2.70 | 1.00 | 0.994 | 0.31; 3.27 |
| 60 - 79 | 1.52 | 0.392 | 0.58; 4.00 | 1.72 | 0.319 | 0.59; 4.96 |
| 80 and above | 1.17 | 0.830 | 0.29; 4.76 | 1.59 | 0.563 | 0.33; 7.64 |
| **Insurance status** (Yes vs no) | 0.73 | 0.538 | 0.28; 1.96 | 0.63 | 0.441 | 0.19; 2.04 |
| **Tobacco use** (Yes vs no) | 1,84 | 0.067 | 0.96; 3.55 | 2.18 | 0.165 | 0.73; 6.55 |
| **Diabetes** (Yes vs no) | 0.34 | 0.083 | 0.10; 1.15 | 0.36 | 0.122 | 0.10; 1.32 |
| **History of tuberculosis** (Yes vs no) | 0.52 | 0.393 | 0.11; 2.35 | 0.67 | 0.550 | 0.18; 2.47 |
| **Use of immunosuppressive drugs** (Yes vs no) | 0.87 | 0.813 | 0.28; 2.71 | 0.31 | 0.246 | 0.04; 2.24 |
| **Contact with TB patient** (Yes vs no) | 1.39 | 0.586 | 0.43; 4.55 | 2.35 | 0.227 | 0.59; 9.34 |
| **History of COVID-19** |  |  |  | Ref. |  |  |
| No COVID-19 infection | Ref. |  |  | 0.67 | 0.498 | 0.21; 2.13 |
| 1-2 months ago | 0.65 | 0.423 | 0.23; 1.85 | 1.06 | 0.885 | 0.48; 2.36 |
| 3 months ago | 0.90 | 0.754 | 0.47; 1.74 | 0.61 | 0.569 | 0.12; 3.28 |
| 4-5 months ago | 0.68 | 0.631 | 0.14; 3.27 |  |  |  |
| **Tuberculosis symptoms** (Yes vs no) |  |  |  |  |  |  |
| Cough | 1.03 | 0.905 | 0.57; 1.88 | 0.95 | 0.878 | 0.47; 1.91 |
| Fatigue | 0.70 | 0.285 | 0.36; 1.35 | 0.59 | 0.216 | 0.25; 1.37 |
| Chest pain | 1.07 | 0.835 | 0.56; 2.07 | 0.66 | 0.386 | 0.26; 1.67 |
| Shortness of breath | 1.89 | 0.054 | 0.99; 3.60 | 2.72 | 0.023 | 1.15; 6.45 |
| Loss of appetite | 0.84 | 0.731 | 0.30; 2.32 | 0.64 | 0.507 | 0.17; 2.38 |
| Unintentional weight loss | 1.11 | 0.842 | 0.39; 3.16 | 1.09 | 0.887 | 0.32; 3.72 |
| Night sweats | 1.62 | 0.387 | 0.54; 4.80 | 1.80 | 0.390 | 0.47; 6.83 |
| Fever | 5.88 | 0.056 | 0.99; 36.14 | 5.94 | 0.107 | 0.68; 51.91 |

**Supplementary Figure S1. Comparative diagnostic performance between QIAreach, TST and QFT-Plus reference standard**

**a. TST-positivity using a 5 mm induration threshold.**


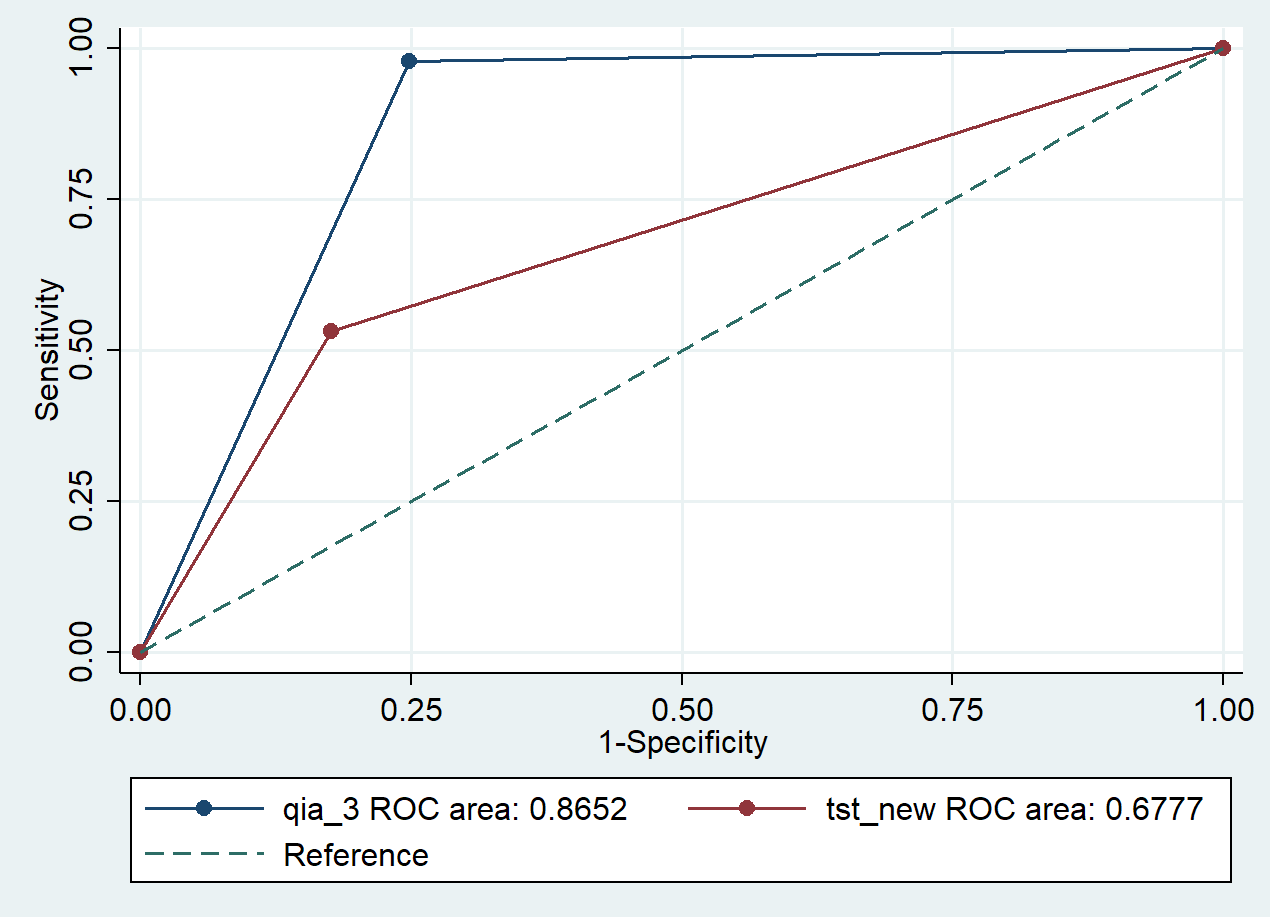


**p < 0.001**

QIAreach

TST (5 mm)

QFT-Plus (ref)

**b. TST-positivity using a 10 mm induration threshold.**

QIAreach

TST (10mm)

QFT-Plus (ref)


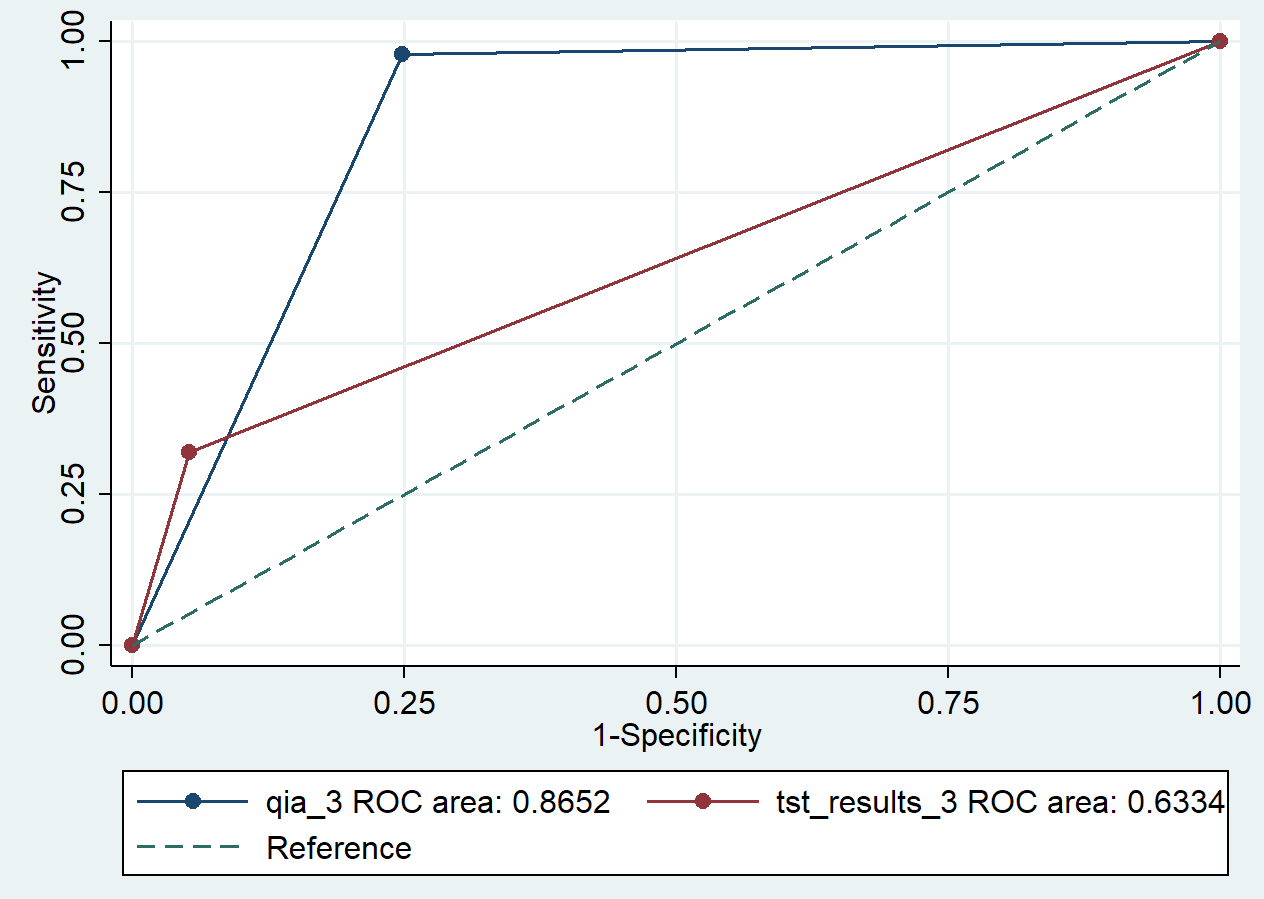


**p < 0.001**

# Legends

**Supplementary Table S1. QFT-Plus result interpretation guidelines**. Manufacturer instructions for QFT-Plus results interpretation.

**Supplementary Table S2.** **QIAreach time-to-result analysis.** Mean time-to-result in seconds disaggregated by QIAreach and QFT-Plus results. Includes standard deviation, median, and interquartile range of reading duration of QIAreach and QFT-Plus were calculated. Mann-Whitney rank-sum tests were used to calculate p-values; p<0.05 was considered statistically significant.

**Supplementary Table S3. Regression analysis of patient covariates associated with discordance between QIAreach QFT TB and QFT-Plus results.** Univariate and multivariate analyses of demographic and clinical factors associated with discordant QIAreach and QFT-Plus results; p<0.05 was considered statistically significant.

**Supplementary Figure S1. Supplementary Figure S1.** **Comparative diagnostic performance between QIAreach, TST and QFT-Plus reference standard.** ROC of QIAreach, TST and QFT-Plus based on a TST-positivity induration threshold of a) 5mm; and b) 10mm.

# STARD Checklist

|  | **Section & Topic** | **No** | **Item** | **Reported on page #** |
| --- | --- | --- | --- | --- |
|  |  |  |  |  |
|  | **TITLE OR ABSTRACT** |  |  |  |
|  |  | **1** | Identification as a study of diagnostic accuracy using at least one measure of accuracy  (such as sensitivity, specificity, predictive values, or AUC) | Page 1 (line 1) |
|  | **ABSTRACT** |  |  |  |
|  |  | **2** | Structured summary of study design, methods, results, and conclusions  (for specific guidance, see STARD for Abstracts) | Page 2 |
|  | **INTRODUCTION** |  |  |  |
|  |  | **3** | Scientific and clinical background, including the intended use and clinical role of the index test | Page 3 |
|  |  | **4** | Study objectives and hypotheses | Page 3 (line 33-34) and  Page 7 (line 21-25) |
|  | **METHODS** |  |  |  |
|  | *Study design* | **5** | Whether data collection was planned before the index test and reference standard  were performed (prospective study) or after (retrospective study) | Page 7 (line 21-25) |
|  | *Participants* | **6** | Eligibility criteria | Page 7 (line 30-33) |
|  |  | **7** | On what basis potentially eligible participants were identified  (such as symptoms, results from previous tests, inclusion in registry) | Page 7 (line 30-33) |
|  |  | **8** | Where and when potentially eligible participants were identified (setting, location and dates) | Page 7 (line 27-29) |
|  |  | **9** | Whether participants formed a consecutive, random or convenience series | Page 7 (line 27) |
|  | *Test methods* | **10a** | Index test, in sufficient detail to allow replication | Page 3 (line 26-33) and  Page 8 (line 5-26) |
|  |  | **10b** | Reference standard, in sufficient detail to allow replication | Page 3 (line 26-33),  Page 8 (line 5-26) and supplementary methods |
|  |  | **11** | Rationale for choosing the reference standard (if alternatives exist) | Page 3 (line 20-23) |
|  |  | **12a** | Definition of and rationale for test positivity cut-offs or result categories  of the index test, distinguishing pre-specified from exploratory | Page 8 (line 13-26) |
|  |  | **12b** | Definition of and rationale for test positivity cut-offs or result categories  of the reference standard, distinguishing pre-specified from exploratory | Page 8 (line 13-26) and supplementary methods |
|  |  | **13a** | Whether clinical information and reference standard results were available  to the performers/readers of the index test | Page 7 (line 21) |
|  |  | **13b** | Whether clinical information and index test results were available  to the assessors of the reference standard | Page 7 (line 21) |
|  | *Analysis* | **14** | Methods for estimating or comparing measures of diagnostic accuracy | Page 8 (line 27-37) |
|  |  | **15** | How indeterminate index test or reference standard results were handled | Page 4 (line 1-2) |
|  |  | **16** | How missing data on the index test and reference standard were handled | Page 8 (line 39-40) |
|  |  | **17** | Any analyses of variability in diagnostic accuracy, distinguishing pre-specified from exploratory | Page 8 (line 27-37) |
|  |  | **18** | Intended sample size and how it was determined | Page 7 (line 34) to  Page 8 (line 4) |
|  | **RESULTS** |  |  |  |
|  | *Participants* | **19** | Flow of participants, using a diagram | Page 3 (line 36) to  Page 4 (line 2) and Figure 1 |
|  |  | **20** | Baseline demographic and clinical characteristics of participants | Page 4 (line 2-8) |
|  |  | **21a** | Distribution of severity of disease in those with the target condition | Page 4 (line 2-8) |
|  |  | **21b** | Distribution of alternative diagnoses in those without the target condition | Page 4 (line 2-8) |
|  |  | **22** | Time interval and any clinical interventions between index test and reference standard | Page 8 (line 5-12) |
|  | *Test results* | **23** | Cross tabulation of the index test results (or their distribution)  by the results of the reference standard | Page 4 (line 9-19) and Table 2 |
|  |  | **24** | Estimates of diagnostic accuracy and their precision (such as 95% confidence intervals) | Page 4 (line 9) to  Page 5 (line 9) |
|  |  | **25** | Any adverse events from performing the index test or the reference standard | Page 4 (line 2-3) |
|  | **DISCUSSION** |  |  |  |
|  |  | **26** | Study limitations, including sources of potential bias, statistical uncertainty, and generalisability | Page 6 (line 34-42) and  Page 7 (line 1-8) |
|  |  | **27** | Implications for practice, including the intended use and clinical role of the index test | Page 5 (from line 17) to Page 6 (line 33) |
|  | **OTHER INFORMATION** |  |  |  |
|  |  | **28** | Registration number and name of registry | Page 9 (line 3-4) |
|  |  | **29** | Where the full study protocol can be accessed | Page 12 (line 13-17) |
|  |  | **30** | Sources of funding and other support; role of funders | Page 12 (line 18-22) |
|  |  |  |  |  |
